# Genomic Variants in *NEURL*, *GJA1* and *CUX2* Significantly Increase Genetic Susceptibility to Atrial Fibrillation

**DOI:** 10.1038/s41598-018-21611-7

**Published:** 2018-02-19

**Authors:** Pengxia Wang, Weixi Qin, Pengyun Wang, Yufeng Huang, Ying Liu, Rongfeng Zhang, Sisi Li, Qin Yang, Xiaojing Wang, Feifei Chen, Jingqiu Liu, Bo Yang, Xiang Cheng, Yuhua Liao, Yanxia Wu, Tie Ke, Xin Tu, Xiang Ren, Yanzong Yang, Yunlong Xia, Xiaoping Luo, Mugen Liu, He Li, Jingyu Liu, Yi Xiao, Qiuyun Chen, Chengqi Xu, Qing K. Wang

**Affiliations:** 1 Key Laboratory of Molecular Biophysics of the Ministry of Education, Cardio-X Center, College of Life Science and Technology and Center for Human Genome Research, Huazhong University of Science and Technology, Wuhan, P.R. China; 2 School of Life Science and Engineering, Graduate School, Lanzhou University of Technology, Lanzhou, P.R. China; 3 Department of Clinical Laboratory, Liyuan Hospital, Tongji Medical College, Huazhong University of Science and Technology, Wuhan, P.R. China; 4 Wuhan Children’s Hospital, Tongji Medical College, Huazhong University of Science and Technology, Wuhan, P.R. China; 5 Department of Cardiology, First Affiliated Hospital of Dalian Medical University, Dalian, P.R. China; 6 Department of Cardiology, People’s Hospital, Wuhan University, Wuhan, P.R. China; 7 Department of Cardiology, Union Hospital, Tongji Medical College, Huazhong University of Science and Technology, Wuhan, P.R. China; 8 Department of Cardiology, the First Affiliated Hospital of Wuhan City, Wuhan, P.R. China; 9 Tongji Medical College, Huazhong University of Science and Technology, Wuhan, P.R. China; 10 College of Physics, Huazhong University of Science and Technology, Wuhan, P.R. China; 11 Center for Cardiovascular Genetics, Department of Molecular Cardiology, Lerner Research Institute, Cleveland Clinic, Cleveland, Ohio USA; 12 Department of Molecular Medicine, Department of Genetics and Genome Sciences, Case Western Reserve University, Cleveland, Ohio USA

## Abstract

Atrial fibrillation (AF) is the most common arrhythmia. In 2014, two new meta-GWAS identified 5 AF loci, including the *NEURL* locus, *GJA1* locus, *CAND2* locus, and *TBX5* locus in the European ancestry populations and the *NEURL* locus and *CUX2* locus in a Japanese population. The *TBX5* locus for AF was reported by us in 2013 in the Chinese population. Here we assessed the association between AF and SNPs in the *NEURL*, *GJA1*, *CAND2* and *CUX2* loci in the Chinese Han population. We carried out a large case-control association study with 1,164 AF patients and 1,460 controls. Significant allelic and genotypic associations were identified between *NEURL* variant rs6584555 and *GJA1* variant rs13216675 and AF. Significant genotypic association was found between *CUX2* SNP rs6490029 and AF. No association was found between *CAND2* variant rs4642101 and AF, which may be due to an insufficient power of the sample size for rs4642101. Together with our previous findings, seven of fifteen AF loci (<50%) identified by GWAS in the European ancestry populations conferred susceptibility to AF in the Chinese population, and explained approximately 14.5% of AF heritability. On the other hand, two AF loci identified in the Japanese population were both replicated in the Chinese population.

## Introduction

Atrial fibrillation (AF) is the most common sustained cardiac arrhythmia with an incidence rate of 1–2% in the general population^1,2^. AF is characterized by fast and irregular abnormal atrial electrophysiological activities, which can lead to >15% of strokes, blood clots and heart failure and increases the rate of sudden death^3–5^. AF is caused by genetic factors, environmental factors and interactions among these factors^6–8^. The heritability of AF is 0.62^9^. Mutations in ion channels such as KCNQ1 and Na_v_1.5 and non-ion channels such as NUP155 and ANF are rare, but can cause AF in isolated AF families^10,11^. On the other hand, genome-wide association studies (GWAS) have been effective in identification of common single nucleotide polymorphisms (SNPs) that increase risk of AF. Early series of GWAS and meta-GWAS in European ancestry populations identified 10 AF-susceptibility loci, including SNPs rs2200733 and rs10033464 near *PITX2c* gene, rs2106261 and rs7193343 in *ZFHX3*, rs13376333 in *KCNN3*, rs593479 in *PRRX1*, rs3807989 in *CAV1*, rs6479562 in *C9orf3*, rs10824026 in *SYNPO2L*, rs1152591 in *SYNE2*, rs7164883 located in *HCN4* and rs2040862 in *WNT8A*^12–16^. In 2014, meta-GWAS in the European ancestry populations identified additional AF susceptibility variants, including *NEURL* SNPs rs12415501, *CAND2* SNP rs4642101, *GJA1* SNP rs13216675 and *TBX5* SNP rs10507248^17^. Also in 2014, GWAS in a Japanese population identified two risk variants for AF, including *NEURL* SNP rs6584555 and *CUX2* SNP rs6490029^17^.

To date, no GWAS were reported for AF in the Chinese population despite the fact that there are over 10 million AF patients in China. Our group previously analyzed the potential association between AF and the 10 AF loci identified in the early series of GWAS and meta-GWAS in the European ancestry populations. We found that three of them, including the *PITX2c*, *ZFHX3* and *CAV1* loci, showed significant association with AF in the Chinese Han population, but other loci were not replicated in the Chinese Han population^18–20^. For the *TBX5* locus, we reported in 2013 that a genomic variant in *TBX5*, rs3825214, showed a significant association with AF in the Chinese population^21^. In this study, we assessed association between AF with other meta-GWAS SNPs identified in European ancestry populations and the Japanese population, including SNP rs6584555 in *NEURL*, rs13216675 near *GJA1*, rs4642101 in *CAND2* and rs6490029 in *CUX2*, in the Chinese GeneID population. We identified significant allelic and genotypic association between *NEURL* rs6584555 and *GJA1* SNP rs13216675 and AF, significant genotypic association between *CUX2* SNP rs6490029 and AF, but no association between *CAND2* SNP rs4642101 and AF.

## Results

### Significant allelic association between *GJA1* SNP rs13216675 and *NEURL* SNP rs6584555 and AF

We carried out a case control association study for AF with four SNPs, including SNP rs4642101 within the *CAND2* gene on chromosome 3p25.2, rs13216675 close to the *GJA1* gene on chromosome 6q22.3, rs6584555 near the *NEURL* gene on chromosome 10q24.33 and rs6490029 within the *CUX2* gene on chromosome 12q24.11. Our study population included 1,164 AF patients and 1,460 non-AF controls from the Chinese Han GeneID population. The average age of the case group was 2.6 years younger than the control group (61.27 ± 11.33 vs. 63.8 ± 13.54, *P* < 0.01). The other characteristics of the case group and the control group are summarized in Table 1. In the control population, the genotypic frequencies for all four SNPs did not deviate from the Hardy-Weinberg equilibrium (*P* > 0.01). The minor allele frequency (MAF) of each SNP in our control population is similar to the data for the Chinese Han population from the HapMap database (Table 2).

**Table 1:** Clinical and demographical characteristics of study subjects.

Characteristics	AF Cases (n = 1,164)	Controls (n = 1,460)	P*
Age (years, mean ± SD)	61.27 ± 11.33	63.8 ± 13.54	<0.01
Male (%)	46.37%	42.18%	0.03
Coronary artery disease (CAD) (%)	24.32%	33.60%	<0.01
Hypertension (HTN) (%)	44.31%	48.62%	0.14
Type 2 diabetes (DM) (%)	14.86%	14.26%	0.31

Data are shown as mean +/− standard deviation (SD) for quantitative variables and percentage (%) for qualitative variables.

*The differences between cases and controls for qualitative variables such as gender, hypertension, type 2 diabetes and CAD were analyzed by a Chi-square (χ^2^) test. The difference for quantitative variables such as means of age was analyzed with a student *t* test.

AF, atrial fibrillation.

**Table 2:** Allelic association analysis between rs4642101, rs13216675, rs6584555 and rs6490029 and AF in the Chinese Han population.

SNP	Chromosomal Position (hg19)	*P*_*hwe*_	Risk Allele	Risk Allele Frequency (cases vs. controls)	Without Adjustment		After Adjustment		Bonferroni correction
					*P*_*obs*_	*OR* *(95%CI)*	*P*_*adj*_	*OR (95%CI)*	*P*
rs4642101 (*CAND2*)	Chr3: 12842223	0.15	G	0.29/0.28	0.23	1.09 (0.95–1.24)	0.19	0.9 (0.79–1.05)	0.57
rs13216675 (near the *GJA1*)	Chr6: 122452329	0.03	T	0.66/0.61	3.9 × 10^−3^	1.2 (1.06–1.37)	0.01	1.19 (1.04–1.35)	0.04
rs6584555 (*NEURL*)	Chr10: 105299611	0.08	C	0.18/0.14	5.08 × 10^−5^	1.38 (1.18–1.62)	9.06 × 10^−5^	1.39 (1.18–1.64)	3.62 × 10^−4^
rs6490029 (*CUX2*)	Chr12: 111698457	0.10	A	0.74/0.72	0.22	0.92 (0.8–1.1)	0.54	1.05 (0.90–1.20)	0.95

*P*_*hwe*_, *P* value for Hardy-Weinberg equilibrium (HWE) tests using PLINK version 1.07 in controls;

*P*_*obs*_, *P* value for association before adjusting for covariates by 2 × 2 contingence table χ^2^ tests using PLINK version 1.07;

*P*_*adj*_, *P* value for association after adjusting for covariates of sex, age, HTN, CAD and DM by multiple logistic regression analysis using SPSS v17.0;

*OR*, odds ratio;

95% CI, 95% confidential interval.

The *GJA1* SNP rs13216675 showed significant association with AF (observed *P*_*obs*_ = 3.9 × 10^−3^, OR = 1.2) (Table 2). After adjusting for covariates of age, gender, hypertension (HTN), diabetes mellitus (DM) and coronary artery disease (CAD), the association remained significant (*P*_*adj*_ = 0.01, OR = 1.19) (Table 2). The common allele T of SNP rs13216675 is the risk allele in the Chinese Han population (Table 2). The significant association between SNP rs13216675 and AF remained after adjusting for multiple testing with Bonferroni correction (corrected *P* = 0.04) (Table 2).

The *NEURL* SNP rs6584555 showed significant association with AF (*P*_*obs*_ = 5.08 × 10^−5^, OR = 1.38) (Table 2). After adjusting for covariates of age, gender, HTN, DM and CAD, the association remained significant (*P*_*adj*_ = 9.06 × 10^−5^, OR = 1.39) (Table 2). The minor allele C of SNP rs6584555 is the risk allele in the Chinese Han population (Table 2). The significant association between SNP rs6584555 and AF remained after adjusting for multiple testing with Bonferroni correction (corrected *P* = 3.62 × 10^−4^) (Table 2).

The two remaining SNPs, *CAND2* SNP rs4642101 and *CUX2* SNP rs6490029 did not show significant allelic association with AF in the Chinese Han population before or after adjustment for covariates (*P*_*obs*_ and *P*_*adj*_ > 0.05) (Table 2).

### Significant genotypic association between *NEURL* SNP rs6584555, *GJA1* rs13216675 and *CUX2* SNP rs6490029 and AF

We also performed the case control association analysis for genotypic frequencies, which may pinpoint potential genetic models under which a significant association is found for a genetic variant in contrast to allelic association analysis. We analyzed the genotypic association for each SNP under three common genetic models: an additive model, a dominant model, or a recessive model. The results are summarized in Table 3.

**Table 3:** Genotypic association analysis between rs4642101, rs6584555, rs13216675 and rs6490029 and AF under three different genetic models.

Model*	Genotypes (AA/AB/BB)		Without Adjustment		Adjustment		Bonferroni correction
	Cases	Controls	*P*_*obs*_	*OR (95%CI)*	*P*_*adj*_	*OR (95%CI)*	*P*
**rs4642101**							
Additive	502/392/95	620/449/100	0.49	n.a	0.18	0.91 (0.8–1.04)	0.55
Dominant	502/487	620/549	0.29	1.10 (0.93–1.30)	0.26	0.9 (0.76–1.1)	0.70
Recessive	894/95	1069/100	0.35	1.14 (0.86–1.53)	0.27	0.85 (0.62–1.14)	0.72
**rs13216675**							
Additive	124/471/460	177/476/430	6.72 × 10^–3^	n.a	0.01	1.19 (1.04–1.35)	0.04
Dominant	124/931	177/906	2.28 × 10^−3^	1.47 (1.15–1.88)	3.04 × 10^−3^	1.49 (1.14–1.92)	0.01
Recessive	595/460	653/430	0.07	1.17 (0.99–1.4)	0.14	0.87 (0.72–1.05)	0.45
**rs6584555**							
Additive	737/294/50	952/290/32	4.03 × 10^−4^	n.a	4.85 × 10^−5^	1.41 (1.19–1.67)	1.94 × 10^−4^
Dominant	737/344	952/322	4.38 × 10^−4^	1.38 (1.15–1.65)	4.26 × 10^−4^	1.43 (1.16–1.72)	1.70 × 10^−3^
Recessive	1031/50	1242/32	5.3 × 10^−3^	1.89 (1.2–2.96)	1.51 × 10^−3^	2.32 (1.37–3.85)	6.03 × 10^−3^
**rs6490029**							
Additive	58/412/530	97/423/578	0.02	n.a	0.54	0.96 (0.83–1.1)	0.95
Dominant	58/942	97/1001	7.97 × 10^−3^	1.57(1.12–2.2)	8.28 × 10^−3^	1.61(1.12–2.27)	0.04
Recessive	470/530	520/578	0.87	1.01(0.86–1.2)	0.65	1.04 (0.87–1.25)	0.96

*Additive model = AA/AB/BB; dominant model = AA + AB/BB; recessive model = AA/AB + BB;

*P*_*obs*_, *P* value for association before adjusting for covariates by 2 × 2 contingence table χ^2^ test using PLINK version 1.07;

*P*_*adj*_, *P* value for association after adjusting for covariates of sex, age, HTN, CAD and DM by multiple logistic regression analysis using SPSS v17.0;

*OR*, odds ratio;

95% CI, 95% confidential interval.

Significant genotypic association was identified between *NEURL* SNP rs6584555 and AF under all three models, although most significant associations were obtained under the dominant and recessive models before and after adjusting for covariates of age, gender, HTN, DM and CAD (*P*_*obs*_ = 4.03 × 10^−4^, *P*_*adj*_ = 4.85 × 10^−5^ under an additive model; *P*_*obs*_ = 4.38 × 10^−4^, *P*_*adj*_ = 4.26 × 10^−4^ under a dominant model; *P*_*obs*_ = 5.3 × 10^−3^, *P*_*adj*_ = 1.51 × 10^−3^ under a recessive model) (Table 3). The significant genotypic association between SNP rs6584555 and AF remained after adjusting for multiple testing with Bonferroni correction (corrected *P* < 0.05) (Table 3).

For *CUX2* SNP rs64990029, although no significant allelic association was found for AF, significant genotypic association was identified for AF under both the additive and the dominant models, but not under the recessive model (*P*_*obs*_ = 7.97 × 10^−3^ under a dominant model; *P*_*obs*_ = 0.02 under an additive model) (Table 3). The significant genotypic association between SNP rs64990029 and AF remained under the dominant model and after adjusting for covariates of age, gender, HTN, DM and CAD (*P*_*adj*_ = 8.28 × 10^−3^) and after further adjusting for multiple testing with Bonferroni correction (corrected *P* = 0.04) (Table 3).

For *GJA1* SNP rs13216675, significant genotypic association was identified for AF under an additive model and a dominant model, but not under a recessive model (*P*_*obs*_ = 6.72 × 10^−3^, *P*_*adj*_ = 0.01 under an additive model; *P*_*obs*_ = 2.28 × 10^−3^, *P*_*adj*_ = 3.04 × 10^−3^ under a dominant model; *P*_*obs*_ = 0.07, *P*_*adj*_ = 0.14 under a recessive model) (Table 3). The significant genotypic association between SNP rs13216675 and AF remained after adjusting for multiple testing with Bonferroni correction (*P* < 0.05) (Table 3).

For *CAND2* SNP rs4642101, similar to the data from allelic association analysis, we did not find any significant genotypic association with AF in the Chinese Han population.

### Estimation of AF heritability explained by SNPs significantly associated with AF in the Chinese population

For the three SNPs showing significant association with AF in the Chinese Han population (*GJA1* SNP rs13216675, *NEURL* SNP rs6584555 and *CUX2* SNP rs6490029), we estimated the heritability of AF explained by each of them. As shown in Table 4, *GJA1* SNP rs13216675, *NEURL* SNP rs6584555 and *CUX2* SNP rs6490029 explained 1.8%, 3.7% and 2.6% of AF heritability, respectively. Together, these three variants explained approximately 8.1% of AF heritability. Previously, we reported three other SNPs which also showed significant association with AF in the Chinese Han population, including SNP rs2200733 on 4q25 and near *PITX2*, rs2106261 on *ZFHX3* locus and rs3807989 on *CAV1*^18–20^. SNP rs2200733, rs2106261 and rs3807989 explained about 6.4% of AF heritability (1.8% for rs2200733, 1.7% for rs2106261 and 2.9% for rs3807989). Together, these six SNPs explained 14.5% of AF heritability.

**Table 4:** Estimation of AF heritability explained by SNPs showing significant association in the Chinese Han population.

Locus	AF heritability explained
rs13216675(near the *GJA1*)	1.8%
rs6584555(*NEURL*)	3.7%
rs6490029(*CUX2*)	2.6%
rs2200733 (near *PITX2*)	1.8%
rs2106261 (*ZFHX3*)	1.7%
rs3807989 (*CAV1*)	2.9%
**Total**	**14.5%**

## Discussion

In this study, we analyzed four genomic variants associated with AF in either the European ancestry populations or the Japanese population for their association with AF in the Chinese Han population. These variants include *NEURL* SNP rs6584555, *GJA1* SNP rs13216675, *CUX2* SNP rs6490029 and *CAND2* SNP rs4642101. Our study population consisted of 1,164 AF patients and 1,460 non-AF controls. Three of the four loci, the *NEURL* locus, *GJA1* locus and *CUX2* locus, were successfully replicated in the Chinese population (Tables 2 and 3). The *CAND2* locus was not replicated in the Chinese population (Tables 2 and 3), which may be due to an insufficient power of the sample size for this variant.

The 2014 meta-GWAS in the European ancestry populations reported four loci for AF, including *NEURL* (rs12415501), *GJA1* (rs13216675), *TBX5* (rs10507248) and *CAND2* (rs4642101). The *TBX5* locus was reported in 2013 by us by studying a Chinese AF population^21^ before the GWAS report. For the three remaining loci, the *NEURL* and *GJA1* loci were significantly associated with AF in the Chinese population, whereas the *CAND2* locus did not show any significant association with AF (Tables 2 and 3). Previously, we showed that only three of the 10 AF GWAS loci identified in the European ancestry populations before 2014 were significantly associated with AF in the Chinese populations^18–20^. Interestingly, the two AF loci reported in the 2014 GWAS in a Japanese population, namely *NEURL* SNP rs6584555 and *CUX2* SNP rs6490029, were both replicated in the Chinese population (Tables 2 and 3). This may be related to the fact that the evolution distance between the Japanese population and the Chinese population is closer that that between the European ancestry populations and the Chinese population.

Our study has a limitation. Our study population of 1,164 AF patients and 1,460 non-AF controls has a sufficient power of 97% and 91% for genomic variants rs6584555 in *NEURL* and rs6490029 in *CUX2*, respectively. However, its power for rs13216675 near *GJA1* and rs4642101 in *CAND2* was 0.40 and 0.39, respectively. Therefore, lack of association between rs4642101 in *CAND2* and AF may be due to the small sample size. Future studies with larger AF case control populations may be needed to further clarify the association between rs4642101 in *CAND2* and AF in the Chinese Han population.

In conclusion, we found significant associations between AF and *NEURL* SNP rs6584555, *GJA1* SNP rs13216675 and *CUX2* SNP rs6490029, but not *CAND2* SNP rs4642101. Together with our earlier reports, we show that among the 15 GWAS loci for AF reported in the European ancestry populations and Japanese population, seven loci (*PITX2c*, *ZFHX3*, *CAV1*, *NEURL*, *GJA1*, *TBX5* and *CUX2* loci) also confer a significant risk of AF in the Chinese Han population. Our findings provide an important understanding of the detailed genomic landscape for AF susceptibility in the Chinese Han population. Our data also suggest that although the European ancestry populations share some common susceptibility loci for AF with the Chinese population, different populations may contain their own unique susceptibility loci for AF.

## Materials and Methods

### Study subjects

The study subjects for this study were from the large GeneID database, which has over 80,000 study subjects with cardiovascular diseases in the Chinese Han population^22–27^. To minimize stratification of population heterogeneity, only study subjects of Han ethnic origin (by self-description) were included. A total of 2,624 subjects were enrolled into this study, including the case group with 1,164 AF patients and the control group with 1,460 non-AF individuals. AF was diagnosed by at least two expert cardiologists based on the criteria of the ACC/AHA/ESC AF guidelines^28^. The control group includes study subjects without AF based on their ECG data and clinical history. The exclusion criteria for both cases and controls include other types of cardiac arrhythmias, ischemic stroke, valvulopathies, structural heart defects, cardiomyopathies and a left ventricular ejection fraction of <50% by electrocardiograms (ECGs), echocardiography, magnetic resonance imaging (MRI) and computed tomography (CT)^19,29^.

This study was approved by the ethics committee on human subject research at Huazhong University of Science and Technology and other appropriate local ethics committees on human subject research. This study is conformed to the guidelines set forth by the Declaration of Helsinki. Written informed consent was obtained from the participants.

### SNP genotyping

Human genomic DNA was extracted from peripheral blood samples using the Wizard Genomic DNA Purification Kit as described previously by us^30^.

Each SNP was genotyped using a Rotor-Gene 6000 High Resolution Melt system as described by us previously^31,32^. The HRM technology is based on the different molecular physical properties of DNA molecules on the fragment length, GC content and GC distribution, which makes DNA molecules with different genotypes (two different homozygotes and the heterozygote) have different shapes and positions of its dissolution curves when heated at different temperatures. The three different genotypes for a genomic variant can then be distinguished based on their different dissolution curves. The polymerase chain reactions (PCR) for genotyping was performed in a 25 μl mixture with 2.5 μl of 10 × PCR buffer, 10 mM dNTP (0.5 μl), 25 mM Mg^2+^ (1.5 μl), 5 pmol of each primer, 25 ng of genomic DNA and 0.7 μl of 5 mM SYTO9. PCR was performed on an ABI9700 System (Applied Biosystems, Foster City, CA) with a thermal profile of 95 °C for 5 minutes, 40 cycles of 95 °C for 15 seconds, 60.3 °C or other appropriate annealing temperatures for 15 seconds and 72 °C for 20 seconds and 72 °C for 10 minutes. Primers for PCR are listed in Table 5. PCR products were directly genotyped using the high resolution melting (HRM) analysis on a Rotor-Gene 6000 System (Corbett Life Science, Australia). DNA samples from 100 study subjects were randomly selected for each SNP for direct Sanger sequencing analysis and the sequencing data completely matched the HRM genotyping data.

**Table 5:** Sequences for primers used for HRM genotyping and direct Sanger sequencing analysis.

SNP	HRM Primers	Sequencing Primers
**rs4642101**		
Forward primer	ggggagagggcagccacaac	ggattgtaggccccgttgta
Reverse primer	gcaggagaatcacttgaacccagg	tcgccagatcacttaaggtcag
**rs13216675**		
Forward primer	gagattagaagagttggattcccc	cctggcaaatgaaagacgtaca
Reverse primer	gcagaccaggaagtattgagt	tgacgaactttgtggcagacc
**rs6584555**		
Forward primer	agaattgttgggtggactttga	gagcgtgcaatgtgtccaatgaa
Reverse primer	cccacattccaggcaagaaa	agggacctgggcttctttcatctt
**rs6490029**		
Forward primer	ccggtggctgccttattg	acccatctcagtttgaaatcgt
Reverse primer	accccctactttcccttcatg	ctggggtctgaggaaaggc

HRM, high-resolution melting; SNP, single-nucleotide polymorphism.

### Statistical analysis

We used PLINK version 1.07 to perform the Hardy–Weinberg linkage equilibrium test in the control group as described by us previously^18,27,33–35^. Pearson 2 × 2 and 2 × 3 contingency table χ^2^ tests were performed with SPSS (version 17.0; SPSS, Inc., Chicago, IL) to analyze allelic association and genotypic association, respectively and to compute odds ratios (ORs) and corresponding 95% confidential intervals (CIs). Multiple logistic regression analysis was used to adjust significant covariates of age, gender, hypertension (HTN), coronary artery disease (CAD) and diabetes mellitus (DM) for AF using SPSS (version 17.0; SPSS, Inc., Chicago, IL).

We estimated the heritability of AF explained by each significant SNP using the multifactorial liability threshold model based on OR estimates using the R package as described previously^36^. The computation of heritability was based on the frequency of the risk allele, relative risk of one risk allele (Aa) over that of no risk allele (aa) (OR: Aa/aa), relative risk of two risk alleles (AA) over that of no risk allele (aa) (OR:AA/aa) and the overall prevalence rate of AF in the population. We assumed a disease prevalence estimate of 0.73% for AF in the Chinese population.

Statistical power analysis of the study population was conducted using program PS (Power and Sample size Calculations, version 3.0.43)^37^. For power analysis, we utilized the reported OR values in the European ancestry populations (*GJA1* SNP rs1321667 and *CAND2* SNP rs4642101) or the Japanese population (*NEURL* SNP rs6584555 and *CUX2* SNP rs6490029)^16,17^, the minor allele frequencies for the studied variants in the Chinese population from the HapMap database and a type I error of 0.05.
